# Supplementary material for: Predictive Factors and Risk Assessment for Hospitalization in Chest Pain Patients Admitted to the Emergency Department
Source: Diagnostics (Basel). 2024 Dec 5;14(23):2733. doi: 10.3390/diagnostics14232733 (PMC11640250; doi:10.3390/diagnostics14232733)
Supplement: Supplementary file 1 [file diagnostics-14-02733-s001.zip › diagnostics-3307740-supplementary.pdf]

Table S1. Factors inserted for multivariate logistic regression analysis in Table 7.

Risk Factors for Hospitalization	OR (95%CI)	P
Group (reference below 65)		0.619
Age 65–74	1.609 (0.612–4.230)	0.335
Age 75–84	1.650 (0.524–5.192)	0.392
Age 85+	0.958 (0.225–4.077)	0.954
Gender (female)	0.991 (0.562–1.747)	0.974
Primary residence—nursing home	0.505 (0.190–1.343)	0.171
Nurse shift (23:00–7:00)		0.151
15:00–23:00	0.575 (0.307–1.078)	0.084
07:00–15:00	0.572 (0.249–1.312)	0.187
IHD	0.873 (0.441–1.725)	0.695
CRF	2.279 (0.695–7.477)	0.174
s/p CVA/TIA	2.490 (0.635–9.762)	0.190
Arrhythmia	0.985 (0.433–2.241)	0.971
Hyperlipemia	3.483 (1.809–6.706)	<0.001
Hypertension	1.575 (0.747–3.324)	0.233
Diabetes	0.907 (0.466–1.763)	0.773
Polypharmacy	0.922 (0.401–2.118)	0.848
Fatigue	1.663 (0.809–3.419)	0.167
Diaphoresis	8.574 (2.522–29.155)	<0.001
Dyspnea	2.758 (1.402–5.426)	0.003

Abbreviations: OR, odds ratio; CI, confidence interval; IHD, ischemic heart disease; CRF, chronic renal failure; s/p CVA TIA, status after cerebrovascular accident/transient ischemic attack; P, probability value.
